# Multiple-locus variable-number tandem repeat analysis of *Salmonella* Enteritidis isolates from human and non-human sources using a single multiplex PCR

**DOI:** 10.1111/j.1574-6968.2007.00875.x

**Published:** 2007-08-10

**Authors:** Seongbeom Cho, David J Boxrud, Joanne M Bartkus, Thomas S Whittam, Mahdi Saeed

**Affiliations:** 1 Department of Large Animal Sciences and Epidemiology, and National Food Safety and Toxicology Center, Michigan State University East Lansing, MI, USA; 2 Department of Large Animal Sciences and Epidemiology, and National Food Safety and Toxicology Center, Minnesota Department of Health St Paul, MN, USA

**Keywords:** *Salmonella* Enteritidis, MLVA, VNTR, PFGE, molecular epidemiology

## Abstract

Simplified multiple-locus variable-number tandem repeat analysis (MLVA) was developed using one-shot multiplex PCR for seven variable-number tandem repeats (VNTR) markers with high diversity capacity. MLVA, phage typing, and PFGE methods were applied on 34 diverse *Salmonella* Enteritidis isolates from human and non-human sources. MLVA detected allelic variations that helped to classify the *S*. Enteritidis isolates into more evenly distributed subtypes than other methods. MLVA-based *S*. Enteritidis clonal groups were largely associated with sources of the isolates. Nei's diversity indices for polymorphism ranged from 0.25 to 0.70 for seven VNTR loci markers. Based on Simpson's and Shannon's diversity indices, MLVA had a higher discriminatory power than pulsed field gel electrophoresis (PFGE), phage typing, or multilocus enzyme electrophoresis. Therefore, MLVA may be used along with PFGE to enhance the effectiveness of the molecular epidemiologic investigation of *S*. Enteritidis infections.

## Introduction

*Salmonella* infections are estimated to cause 1.4 million cases and more than 500 deaths a year in the United States (Mead *et al.*, 1999) and economic losses caused by salmonellosis are projected to be from $0.5 to $2.3 billion per year. Currently, *Salmonella enterica* serovar Enteritidis is the most common *Salmonella* serotype encountered in foodborne salmonellosis in Europe and is one of the most common *Salmonella* serotypes recovered from human infections in North America. Phage typing is the most common method used for subtyping of *S*. Enteritidis but is available only at a small number of laboratories. However, phage types may be unstable and several *S*. Enteritidis strains do not conform to any of the specific phage types in current use (Rankin & Platt, 1995).

Furthermore, pulsed-field gel electrophoresis (PFGE), which is currently the gold-standard technique for subtyping bacterial isolates associated with common foodborne infections, is laborious, requires precise standardization, and is also of limited effectiveness in subtyping *S*. Enteritidis isolates (Hudson *et al.*, 2001; Liebana *et al.*, 2001; Swaminathan *et al.*, 2001; Lukinmaa *et al.*, 2004; Hyytia-Trees *et al.*, 2006). The changing epidemiology of *S*. Enteritidis infections requires typing methods of high discriminatory power, that are reproducible, stable, easy to interpret, and compare between laboratories. Recently, DNA sequence-based methods such as multilocus sequence typing (MLST) have been commonly used in subtyping of bacteria with high conserved sequences, as genome sequence data are available at GenBank (Lukinmaa *et al.*, 2004; Maiden, 2006). However, MLST is laborious and time-consuming and had limited ability to resolve genetic diversity between *S. enterica* serovars compared with PFGE (Fakhr *et al.*, 2005; Torpdahl *et al.*, 2005).

Recently, multiple-locus variable-number tandem repeat analysis (MLVA) has been proposed as an alternative to PFGE for subtyping of a number of pathogenic bacteria, especially highly clonal groups of microorganisms including *Francisella tularensis*, *Bacillus anthracis*, *Escherichia coli* O157 : H7, *Mycobacterium paratuberculosis*, and *Coxiella burnetii* (Keim *et al.*, 2000; Farlow *et al.*, 2001; Klevytska *et al.*, 2001; Lindstedt *et al.*, 2003b, 2004a, b; Overduin *et al.*, 2004; Ramisse *et al.*, 2004; Svraka *et al.*, 2006). MLVA is based on the detection of short sequence repeats that vary in copy number (i.e. variable number of tandem repeats or VNTR) in the microbial genome at various regions. The VNTR are often highly polymorphic with variation in both the number of repeat units and by sequence heterogeneity among individual units (van Belkum, 1999).

MLVA typing of *S*. Enteritidis has been described previously (Boxrud *et al.*, 2007). The objectives of this report were to describe the optimization of this MLVA to enable performing a one-shot multiplex PCR for seven VNTR markers with high diversity and to compare its discriminatory power with PFGE and phage typing on a diverse group of *S*. Enteritidis isolates.

## Materials and methods

### Bacterial strains

Thirty-four *S*. Enteritidis strains originally isolated from a variety of sources from 1978 to 2004 were used in this study (Table 1). The strains represent the major phage types identified in the United States and include a variety of PFGE profiles, antimicrobial resistance profiles, and distinct multilocus enzyme electrophoresis (MLEE) types. The strains were selected to represent various host and tissue sources including humans, chickens, eggs, mice, cows, wild animals, and the environment (chicken farms). The *S*. Enteritidis strains were phage typed at the National Veterinary Service and CDC laboratories (Ward *et al.*, 1987). These strains were also analyzed for attachment and invasiveness to Hep-2 tissue culture cells, antimicrobial resistance profiling using the NARMS panel of 12 antimicrobials in a ‘Sensititer’ system, and MLEE to screen the isolates for 18 housekeeping enzymes were performed as described earlier (Saeed *et al.*, 2006). PFGE for the isolates was performed at the Michigan Department of Community Health Bureau of Laboratories following the PulseNet standardized protocol (Ribot *et al.*, 2002).

**Table 1:** Phenotypic and Genotypic Characterization of *Salmonella* Enteritidis isolates (*n* = 34) from multiple sources

Isolates	Source	Year	Location	PT	MLEE	Attach	Invasive	Antimicrobial Resistance	PFGE*	
									
									XbaI	BlnI
H266	Human	1995	IN	8	N/A	MRDA	I	N/A	00SenXb.001	00SenBl.001
C145	Chicken	1995	IN	28	N/A	N/A	I	Sensitive	00SenXb.001	00SenBl.001
E39	Egg	1994	IN	13a	3	MSLA	I	Sensitive	00SenXb.001	00SenBl.001
M3	Mouse	1991	IN	8	3	MRLA	I	Sensitive	00SenXb.001	00SenBl.001
M8	Mouse	1991	IN	8	3	MRLA	I	Sensitive	00SenXb.001	00SenBl.001
M9	Mouse	1991	IN	8	3	MRLA	I	Sensitive	00SenXb.001	00SenBl.001
M10	Mouse	1991	IN	8	3	MRLA	I	Sensitive	00SenXb.001	00SenBl.001
H314	Human	1994	IN	8	N/A	MSDA	I	Sensitive	00SenXb.001	00SenBl.001
B2091	Bovine	1991	WA	RDNC	3	MRLA	I	AMP/KAN/STR/TER	00SenXb.001	00SenBl.001
H2	Human	1990	IN	8	N/A	MRLA	I	Sensitive	00SenXb.001	00SenBl.001
H5	Human	1990	IN	8	N/A	MRLA	I	Sensitive	00SenXb.001	00SenBl.001
H11	Human	1990	IN	8	N/A	MRLA	I	Sensitive	00SenXb.001	00SenBl.001
H18	Human	1990	IN	8	N/A	MRLA	I	Sensitive	00SenXb.001	00SenBl.001
H172	Human	1991	IN	8	17	MSLA	NI	Sensitive	00SenXb.001	00SenBl.001
H310	Human	1995	IN	13a	N/A	N/A	NI	Sensitive	00SenXb.001	00SenBl.001
C111	Chicken	1995	IN	13a	N/A	N/A	I	Sensitive	00SenXb.001	00SenBl.001
E2	Egg	1991	PA	8	3	N/A	NI	Sensitive	00SenXb.001	00SenBl.001
E46	Egg	1994	IN	13a	3	N/A	NI	Sensitive	00SenXb.001	00SenBl.001
H8739	Human	2004	WA	RDNC	3	MRLA	I	AMP/SUL/TET/SXT	00SenXb.004	05SEnBl.003
H8780	Human	2004	WA	29a	24	MRLA	I	SUL/TET/SXT	00SenXb.004	05SEnBl.003
Min2842	Mink	1995	WA	4	3	MRLA	I	KAN/STR/SUL	00SenXb.004	00SenBl.003
H33	Human	1997	IN	4	3	MRLA	I	N/A	00SenXb.004	05SenBl.004
H9336	Human	2004	WA	1	3	MSLA	I	SXT	00SenXb.016	05SenBl.005
H31	Human	1997	IN	4	3	MSLA	NI	N/A	02SenXb.001	05SenBl.009
V39	Environment	1991	IN	28	N/A	N/A	NI	Sensitive	02SenXb.007	05SenBl.006
V43	Environment	1991	IN	28	N/A	N/A	I	Sensitive	02SenXb.007	05SenBl.007
C471	Chicken	1978	IN	28	3	MRDA	NI	Sensitive	04SenXb.002	05SenBl.001
C467	Chicken	1978	IN	28	3	MRLA	I	Sensitive	04SenXb.002	05SenBl.001
C478	Chicken	1978	IN	28	3	MRLA	I	Sensitive	04SenXb.002	05SenBl.001
E3	Egg	1991	PA	8	3	MRLA	I	Sensitive	05SenXb.007	00SenBl.001
E75	Egg	1991	PA	Untype	3	N/A	I	Sensitive	05SenXb.007	00SenBl.001
Mu8930	Mule Deer	2004	WA	1b	22	MRLA	I	SUL	05SenXb.008	05SenBl.010
C172	Chicken	1991	OH	13a	N/A	MRLA	NI	Sensitive	05SenXb.009	05SenBl.001
H10	Human	1990	IN	8	N/A	MRLA	I	Sensitive	05SenXB.010	05SenBl.008

* The first two characters in the code denote the year of subtypes identified, the next three characters represent *S*. Enteritidis strains, the next two characters indicate the enzyme used for DNA restriction, and the last three characters mean the pattern number. The pattern designation is based on indistinguishable patterns resolved by either XbaI or BlnI enzyme.

MRDA, mannose-resistant diffuse attachment; MRLA, mannose-resistant localized attachment; MSLA, mannose-sensitive localized attachment; I, invasiveness; NI, noninvasiveness; AMP, ampicillin; KAN, kanamycin; STR, streptomycin; SUL, sulfisoxazole; SXT, trimethoprim-sulfamethoxazole; TET, tetracycline; N/A, not available; RDNC, reactive but does not conform to a known pattern.

### DNA isolation

Strains were cultured overnight at 37°C on tryptic soy agar plates. A generous loopful of each culture was suspended in 200 μL distilled water. For DNA extraction, cell suspensions were heated to 98°C on a heat block for 5 min and then immediately cooled on ice for 5 min. Cell suspensions were centrifuged (20 000 ***g***, 5 min), and supernatants were used as the DNA template for PCR amplification. DNA quantification was measured with a NanoDrop© ND-1000 spectrophotometer (NanoDrop Technologies Inc., Rockland, DE), after which diluted samples (1 : 100–1 : 300 sterile DI water) were prepared and the remainder was frozen at −20°C in 30% glycerol for later use.

### VNTR loci and multiplex PCR

Tandem repeat loci and their primers were based on the genome sequences of *S*. Enteritidis LK5, *S*. Enteritidis PT4, and *S*. Typhimurium LT2 strains as described previously (Boxrud *et al.*, 2007). A primer set for SE7 locus was redesigned in this study to yield a PCR amplicon that can be analyzed with the Beckman 600-bp DNA size marker and that can be separated by capillary electrophoresis under the same condition from amplicons of the other loci. Seven VNTR loci selected for MLVA were amplified in a single multiplex PCR with seven primer sets (SE1, SE2, SE3, SE5, SE7, SE8, and SE9) using forward primers labeled with a WellRED dye (Sigma-Proligo, Boulder, CO) at the 5′ end and nonlabeled reverse primers (Integrated DNA Technologies, Coralville, IA) described in Table 2. A master mix was made for 20 μL reactions containing the following components: 10 μL of 2 × Qiagen multiplex PCR Master mix, 2 μL of MgCl_2_ (25 mM), 2 μL of 10 × primer mix (0.3–1.5 μM per each primer), 3 μL of the dilute DNA template (0.1–1.0 ng μL^−1^), and RNase-free water to a volume of 20 μL. Samples were loaded into a GeneAmp PCR system 9700 (Applied Biosystems). PCR was performed with predenature of 95°C for 15 min, then 35 cycles of 94°C for 30 s, 58°C for 90 s, 72°C for 90 s followed by a final elongation of 60°C for 30 min.

**Table 2:** Tandem repeats and primers for one-shot multiplex PCR amplification of seven VNTR loci for MLVA

Locus	Primer	Primer sequence (5′–3′)	Tandem repeat sequence	Repeat size (bp)	Conc. (μM)	No. of repeats	Allelic diversity*
SE1	Forward Reverse	(D3)AGACGTGGCAAGGAACAGTAG CCAGCCATCCATACCAAGAC	ACCAACT	7	0.10	5–6	0.36
SE2	Forward Reverse	(D4)CTTCGGATTATACCTGGATTG TGGACGGAGGCGATAG	CCGGCAT	7	0.05	5–9	0.63
SE3	Forward Reverse	(D2)CAACAAAACAACAGCAGCAT GGGAAACGGTAATCAGAAAGT	TATTGTTTTCCA	12	0.10	3–4	0.29
SE5	Forward Reverse	(D2)CGGGAAACCACCATCAC CAGGCCGAACAGCAGGAT	ATGGTC	6	0.10	4–11	0.70
SE7†	Forward Reverse	(D4)CCGACCCAATAAGGAG CTTACCGTTGGTAGTTTGTTA	CGGTTTATCCCCGCTGGCGCGGGGAACACA AGCCCCGGCAGCGGTAGCTAAACTAGCACC	60	0.03	5–7	0.34
SE8	Forward Reverse	(D2)TTGCCGCATAGCAGCAGAAGT GCCTGAACACGCTTTTTAATAGGCT	AGCCAAATAAATATATTGGCTTATACTCGT CATACTTCAAGTTGCATGTGCTGCGGCCG CGTTCCCTCACCCCAGTCACTTACTTTA	87	0.15	1–2	0.46
SE9	Forward Reverse	(D4)CGTAGCCAATCAGATTCATCCCGCGTTTGAAACGGGGTGTGGCGCTG	CCATATTCG	9	0.10	2–3	0.25

* Nei's diversity index as 1–Σ (allele frequency)^2^.

† Tandem repeat sequence was variable between *S*. Enteritidis isolates and the percent match of sequence was 64–73% (degenerate repeat).

### Capillary electrophoresis

The PCR products were purified using MultiScreen™ 96-well plates (Millipore, Bedford, MA) with hydrated Sephadex and loaded into a Beckman CEQ 8000 automated DNA sequencer (Beckman-Coulter) according to the manufacturer's suggested protocol. The fragment size for each locus was determined by combining 1.0 μL of PCR product, 38.5 μL sample loading solution, and 0.5 μL of CEQ 600-bp DNA size standard (Beckman-Coulter) to each sample well. The conditions for capillary separation of fragments for all loci included a capillary temperature of 35°C, denature temperature of 90°C, duration of 120 s, injection voltage of 2.0 kV, duration of 30 s, separation voltage of 7.5 kV, and separation duration of 50 min.

### DNA sequencing

To verify the genetic basis of the results from MLVA, the copy number variations of tandem repeats for distinct alleles at all seven VNTR loci were analyzed by direct sequencing. After VNTR amplification using nonfluorescently labeled primers, the PCR products were purified using QIAquick PCR purification kits (QIAGen Inc., Valencia, CA). The forward and reverse strands of the purified PCR amplicons were sequenced using a Beckman CEQ 8000 DNA sequencer (Beckman Coulter, Fullerton, CA) and GenomeLab DTCS Quick Start kit (Beckman Coulter, Fullerton, CA) according to the manufacturer's suggested protocol. After sequences were aligned to create contigs using SeqMan (DNASTAR Inc., Madison, WI), the numbers of tandem repeats were counted using a tandem repeats finder software (accessible at http://tandem.bu.edu/trf/trf.html) (Benson, 1999). The copy numbers were rounded to the nearest integer and entered into the MLVA profiles.

### Diversity and discriminatory power

For the evaluation of the discriminatory capacity of the allelic variation at each VNTR marker, Nei's diversity index was calculated as 1–Σ (allele frequency)^2^.

The probability that another *S*. Enteritidis isolate in the population has the same MLVA type was measured by multiplying the frequency of each allele for seven loci, which assumes that the alleles occur at random in genotypes. Simpson's diversity (1-D) and Shannon's diversity (H′) indices were calculated to evaluate the discriminatory power of subtyping methods as described previously (Boxrud *et al.*, 2007).

### Cluster analysis

After sequencing verification process, the allele scores based on the fragment size were converted into repeat numbers of the seven loci and entered into bionumerics software (Applied-Maths, St-Martens-Latem, Belgium) as character data for cluster analysis. A dendrogram was generated using the categorical coefficient and unweighted pair group method with arithmetic means (UPGMA) of the bionumerics software (version 4.5). This categorical parameter implies that the same weight is given to any multistate character at each locus, whatever the repeat number is (Ramisse *et al.*, 2004). A minimum-spanning tree (MST) was also generated using the categorical coefficient of the software to calculate the distance matrix. In case of equivalent solutions in terms of calculated distance, the highest number of single locus variants (SLVs; in case two types have an equal distance to a linkage position in the tree, the type that has the highest number of SLVs is linked first) associated was used as the priority rule for linking types in the tree. Creation of hypothetical types (missing links) was allowed to introduce hypothetical types as branches of the MST, causing the total spanning of the tree to decrease significantly.

## Results and discussion

The MLVA method has been optimized for the typing of a diverse group of 34 *S*. Enteritidis isolates. Diversity of 34 *S*. Enteritidis isolates is demonstrated by 10 different phage types, several patterns of attachment and invasiveness, and antimicrobial resistance expressed by six isolates, including four isolates with multi-drug resistance (Table 1). MLEE type (ET) 3 was the most common among the 34 *S*. Enteritidis isolates. Ten PFGE patterns were identified using XbaI enzyme while 11 PFGE patterns were identified using BlnI enzyme. Combined patterns of the two enzymes resulted in 13 distinct PFGE genotypes.

**Fig. 1:**
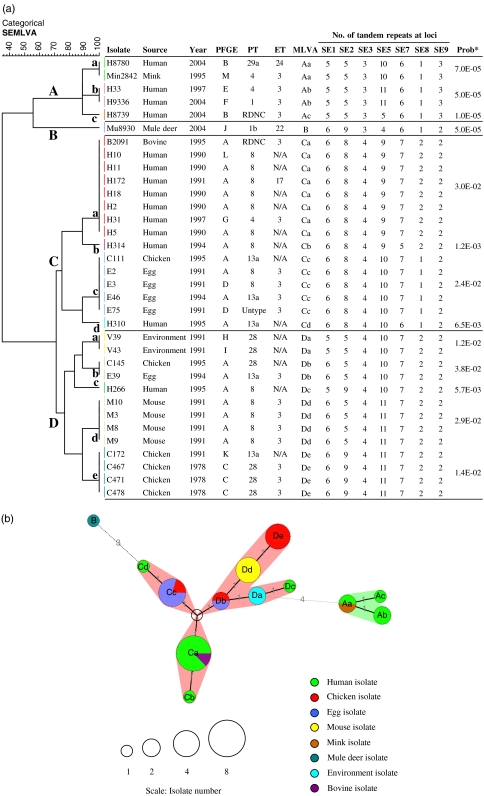
Cluster analysis of the 34 *S*. Enteritidis isolates from multiple sources. (a) Dendrogram of MLVA. Four major clusters or lineages labeled A–D were defined of groups of closely related strains sharing on average ∼75% of the allelic congruence resolved by MLVA genotypes. Two PFGE pattern designations resolved by the use of XbaI and BlnI restriction enzymes, respectively, were combined to produce a cumulative PFGE type. ^*^: Probability that another *S*. Enteritidis isolate in the population has the same MLVA profile was calculated by multiplying frequency of each allele at seven VNTR loci. For example, the probability of 0.038 for isolate E39 resulted from multiplying allele frequency for locus SE1 (26/34=0.76) by similarly calculated frequencies of alleles for the other six loci [SE2 (0.38) × SE3 (0.82) × SE5 (0.38) × SE7 (0.76) × SE8 (0.65) × SE9 (0.85)]. (b) Minimum spanning tree of MLVA. Clonal complexes were created based on maximum neighbor distance of changes at two loci and minimum size of two types. The numbers between MLVA types indicate distances (changes in loci) between two neighboring types. The central circle between clusters C and D represents a hypothetical type as a missing type for which a number of SLVs exist in the data set of the MST. The sizes of the circles depend on the number of samples (their population size). Wedges in circles represent the proportion of *S*. Enteritidis isolates from respective sources.

Tandem repeat sequences and diversity for VNTR loci are shown in Table 2. The genetic diversity estimates for seven loci ranged from 0.25 for SE9 to 0.70 for SE5. MLVA distinguished 13 different types among the 34 *S*. Enteritidis isolates from diverse sources (Fig. 1). These were classified into four main clusters by similarity matrices based on the categorical coefficient and UPGMA (Fig. 1a). In the dendrogram, cluster A is composed mostly of *S*. Enteritidis strains recently isolated from humans. The strains are SLVs at locus SE5. However, based on categorical parameters, these isolates may be considered to belong to the same cluster regardless of copy numbers in this dendrogram (Fig. 1a). Cluster B consists of a single strain isolated from mule deer (resistant to Sulfisoxazole) and is distinct from the lineages with allele differences at loci 2 and 5. Cluster C is composed of nine human clinical isolates, one bovine isolate, four egg isolates, and one chicken isolate. MLVA types Ca and Cb are SLVs at locus SE7 and are very closely related. A major type Ca is also related to double locus variants (DLVs) type Cc that includes isolates from eggs and chicken. Type Cd includes an isolate from human source. Cluster D comprises five types: Da (chicken farm), Db (chicken and egg), Dc (human), Dd (mice), and De (chicken). There are SLVs between types Da and Db and between types Dd and De. Defined *S*. Enteritidis MLVA types appeared to cluster largely by source and year of isolation (Fig. 1a).

The probability that another *S*. Enteritidis isolate with the same MLVA profile based on seven VNTR markers occurs in the population ranged from 0.038 to 0.00001 (Fig. 1a). It was found that MLVA types Ca, Cc, Da, Db, Dd, and De appeared to be clonal groups, which can be more frequently found than other types based on allele frequency at seven VNTR loci among *S*. Enteritidis populations (Fig. 1a). The MLVA type with the highest frequency alleles was Db, which included chicken and egg isolates.

An MST analysis was conducted to develop a model for the evolutionary steps in the divergence of the *S*. Enteritidis MLVA genotypes (Fig. 1b). The MST posits an intermediate clone that is hypothetical and not part of the real data set. MLVA type Db and a hypothetical intermediate type are located in the center of the MST, indicating they may be the evolutionary origin of other types but more *S*. Enteritidis isolates from diversified sources are needed for inclusion into this model for a more valid comparison. Clusters C and D could have branched off from a hypothetical type even though they were clustered together into a single clonal complex in the MST. There are two branches (Da, Dc, and Dd, De) off from type Db that constitute cluster D. Cluster A (Aa, Ab, and Ac) has neighbor distance of changes at four loci from a major central cluster (clusters C and D). Three recent isolates of antimicrobial-resistant *S*. Enteritidis from humans (Aa, Ab, and Ac) and one multidrug-resistant *S*. Enteritidis isolate from mink (Aa) belonged to this cluster. Cluster B is distant in changes at three loci from the major central cluster. A mule deer strain in cluster B is distinct in its unique PFGE, phage type, and MLEE type, suggesting its overall level of genetic divergence. The fact that these isolates were recently obtained from the listed sources may suggest the occurrence of an evolutionary process that affected the profiles of the isolates.

**Fig. 2:**
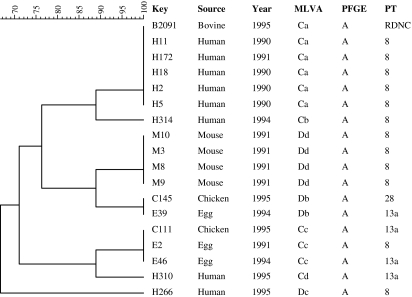
Dendrogram of MLVA based on the 18 *S*. Enteritidis isolates of the same PFGE type. Two major PFGE pattern designations, 00SenXb.001 and 00SenBl.001, resolved by the use of XbaI and BlnI restriction enzymes, respectively, were combined and assigned as a cumulative major PFGE type ‘A’. Seven different MLVA types were identified from 18 *S*. Enteritidis isolates with the identical PFGE types. A major type of MLVA designated as Ca (mostly PT8, predominant PT in US) included five isolates from human sources during 1990–1991 and one bovine isolate in 1995.

MLVA has been reported to produce larger numbers of subtypes of *E. coli* O157 (Noller *et al.*, 2003; Keys *et al.*, 2005; Hyytia-Trees *et al.*, 2006) and *S. enterica* serovar Typhimurium (Lindstedt *et al.*, 2003a) than does PFGE. A previous study on *S*. Enteritidis (Boxrud *et al.*, 2007) demonstrated that MLVA yielded higher discriminatory power than did PFGE when tested using a panel of human clinical isolates of *S*. Enteritidis recently isolated from Minnesota. In the present study, the higher discriminatory power of MLVA for *S*. Enteritidis enabled the resolution of 18 *S*. Enteritidis isolates from diverse sources, but with identical PFGE patterns, into seven MLVA types. These *S*. Enteritidis MLVA types were largely clustered by source of isolation irrespective of their multiple phage types, which can serve as a useful epidemiologic tool in the investigation of *S*. Enteritidis infections (Fig. 2). For example, *S*. Enteritidis isolates from chickens and eggs were clustered together in each of two types (Cc and Db), which may support the widely perceived epidemiological linkage between these two sources. MLVA type Ca, Cb, Cd, and Dc were associated with isolates from human sources and type Dd was associated with mouse *S*. Enteritidis isolates.

Simpson's diversity index and the 95% confidence intervals (95% CI) of subtyping methods were calculated in Table 3. Shannon's diversity index (H′) for MLVA was compared with other subtyping methods. Diversity indices of both Simpson and Shannon's were significantly higher in MLVA than other subtyping methods (Table 3). Although PFGE and MLVA grouped the 34 *S*. Enteritidis isolates into 13 types (Fig. 1a), MLVA had higher diversity indices than PFGE since MLVA types were more-evenly distributed than PFGE types. The number of *S*. Enteritidis isolates for the maximum, third quartile, and median were 8, 4, and 2 for MLVA types and 18, 2, and 1 for PFGE types, respectively.

**Table 3:** Diversity indices of subtyping methods

		Simpson's			Shannon's		
			
Subtyping		*N*	1D	95% CI	H′	*t*-Statistic*	*P* value
MLVA†		34	0.90	0.85–0.95	2.31	–	–
PFGE‡	Both	34	0.72	0.55–0.88	1.82	2.14	0.04
	XbaI	34	0.71	0.55–0.87	1.66	3.14	<0.01
	BlnI	34	0.65	0.47–0.83	1.56	3.23	<0.01
Phage type§		34	0.79	0.68–0.89	1.75	3.30	<0.01
MLEE		21	0.27	0.02–0.52	0.57	7.48	<0.01

* The *t*-test statistic of Shannon's index of diversity (H′) for MLVA was compared with other subtyping methods.

† Diversity indices (both Simpson's and Shannon's) were significantly higher in MLVA than other subtyping methods.

‡ Using one or two restriction enzymes (XbaI, BlnI) in PFGE did not result in significant changes in diversity indices.

§ There was no significant difference in Shannon's index of diversity (H′) between PFGE and phage typing (*P* = 0.78).

Molecular subtyping methods that can be used to relate disease-causing pathogens to their probable sources are needed. PFGE has been the standardized method used in PulseNet, the national molecular subtyping network for the epidemiological investigation of several foodborne pathogens. However, a combination of PFGE and phage typing has been applied for the characterization of *S*. Enteritidis isolates from different sources (Liebana *et al.*, 2001, 2002). PFGE subtyping of *S*. Enteritidis did not result in diverse profiles because the genomes of *S*. Enteritidis are highly similar (Hudson *et al.*, 2001; Liebana *et al.*, 2001). In this study, the previously described MLVA method for *S*. Enteritidis subtyping has been optimized, so that it may be performed as a single multiplex PCR followed by DNA fragment analysis.

In epidemiologic investigations, the sensitivity and effectiveness of a laboratory method, along with other information, to identify the source of infectious agents, are vital if early control and preventive measures against the disease. If the host adaptation leaves a print of particular variation in even one locus of the VNTR, MLVA is more likely to help in tracing back the *S*. Enteritidis to its source than PFGE and phage tying. Because the identification of sources of foodborne pathogens is important for the control of foodborne diseases, the high discriminatory power of MLVA can be of significant value in the epidemiologic investigation of outbreak and sporadic cases of *S*. Enteritidis infection (Boxrud *et al.*, 2007).

Further study may be needed to compare allele distribution or genetic diversity for each locus of *S*. Enteritidis isolates from different sources for tracing purposes. Among the limitations of this study is the relatively small number of *S*. Enteritidis isolates; for example, the small numbers of samples used in an MST-based cluster may not be adequate to construct a valid population modeling.

In conclusion, the MLVA method has been optimized based on a single multiplex PCR using seven VNTR markers with high diversity capacity. Tandem repeat numbers for each locus were verified after the sequencing step. This method can be used along with PFGE to enhance the effectiveness of the molecular epidemiologic investigation of *S*. Enteritidis infections.

## Statement

Reuse of this article is permitted in accordance with the Creative Commons Deed, Attribution 2.5, which does not permit commercial exploitation.
